# Co‐Design of the Structured Personalised Assessment for Reviews After Cancer (SPARC) Intervention

**DOI:** 10.1111/hex.70174

**Published:** 2025-02-06

**Authors:** Rosalind Adam, Lisa Duncan, Sara MacLennan, Louise Locock, Anne E. Kiltie, Leslie Samuel, Peter Murchie

**Affiliations:** ^1^ Academic Primary Care, Institute of Applied Health Sciences University of Aberdeen Aberdeen UK; ^2^ Academic Urology Unit, Institute of Applied Health Sciences University of Aberdeen Aberdeen UK; ^3^ Aberdeen Centre for Evaluation, Institute of Applied Health Sciences University of Aberdeen Aberdeen UK; ^4^ Aberdeen Cancer Centre, Rowett Institute University of Aberdeen Aberdeen UK; ^5^ Oncology Department, School of Medicine, Medical Sciences and Nutrition, Aberdeen Royal Infirmary University of Aberdeen Aberdeen UK

**Keywords:** cancer, chronic disease management, co‐design, multimorbidity, primary care

## Abstract

**Introduction:**

An increasing number of people are living beyond cancer with unmet health needs. The aim of this study was to co‐design a digital intervention to improve health outcomes for people who have completed potentially curative treatment for cancer.

**Methods:**

Two co‐design workshops were held with patients, clinicians (including oncologists, general practitioners and nurses), digital/computing science experts and third‐sector representatives. At workshop one, problems and gaps in care were identified and intervention ideas were generated. At workshop two, a prototype intervention was discussed and refined.

**Results:**

The workshops were attended by 43 people in total: 26 at event one and 23 at event two (six attended both events). Patients valued relationship‐based care and felt supported during hospital treatment. Patients ‘fell off a cliff’ after discharge, and there was consensus that more could be done in primary care to support those living beyond cancer. It was proposed that cancer reviews could be integrated into UK primary care chronic disease management activities. A digital form, the ‘Structured Personalised Assessment for Reviews after Cancer’ (SPARC) tool, was developed to support asynchronous consultations that would cover the breadth of problems and health promotion activities required for high‐quality primary care for cancer. SPARC could also identify those without problems who do not require review.

**Conclusion:**

SPARC has been co‐designed to support brief but comprehensive cancer review consultations between primary care clinicians and their patients. SPARC aligns with best practice guidelines. The next step is to evaluate SPARC with patients and in general practices.

**Patient and Public Contribution:**

Patient and stakeholder engagement was at the centre of this research study. Cancer organisations such as ‘CLAN’ cancer support, Prostate Cancer Scotland and Cancer Research UK helped us to engage with patients. The Aberdeen University Institute of Applied Health Science Patient Public Involvement group were also instrumental in sense‐checking and improving the materials for the second workshop. We plan to involve our patient and carer partners in designing the next stages of our research (including study materials, processes and methods) so that they will be at the centre of evaluating the intervention that they have been instrumental in designing.

## 1 Introduction

The number of people living with and beyond cancer is increasing due to ageing populations and improvements in early detection and treatments [1]. In the United Kingdom, the number of people living with and beyond cancer is expected to grow by approximately 1 million every decade, from 2.1 million in 2010 to 5.3 million in 2040 [2]. In 2022, there were over 18 million people living with or beyond cancer in the United States of America of whom over half were diagnosed within the past 10 years [1]. In Europe, approximately 5% of the population have previously had a cancer diagnosis [3]. Health and social care systems throughout the world must adapt to meet the growing burden of cancer and its sequelae [4].

Healthcare systems are shifting some aspects of cancer aftercare away from hospital oncology clinics towards community‐based self‐monitoring and self‐management [5, 6] but cancer aftercare can create significant work for patients and their caregivers [7]. Governments and policy‐makers expect that digital technologies will improve the efficiency, accessibility and quality of health care [8, 9]. However, without careful design and consideration of patients' needs, digital technologies could also contribute to treatment burden [10].

People living with and beyond cancer can experience a variety of unmet needs including burdensome symptoms such as pain and fatigue, psychological distress, fear of recurrence and financial toxicity [11]. A wide range of digital interventions has been designed to promote self‐management of unmet needs and to promote healthy behaviours after cancer [12]. There is increasing evidence that some interventions can improve symptom control [13] and physical activity levels [14]. The evidence is less clear about whether digital interventions can improve quality of life [15] and there are gaps in the implementation, adoption and scalability of these interventions [16].

One theory‐based multi‐faceted digital intervention, ‘Renewed’, offered bespoke symptom management, psychological and behavioural support to people who had finished treatment for cancer [17]. A randomised controlled trial enroled people who had finished treatment for cancer from United Kingdom general practices and compared ‘Renewed’ against generic online support [18]. There were improvements in global health and symptom management in the intervention group at 12 months but no significant differences in quality of life between the groups at 6 or 12 months [18]. Qualitative process assessment revealed barriers to patient engagement, including low perceived need for the intervention [19]. Some participants described joining the trial ‘to give back’ to others or the scientific community after cancer [19]. Adherence to digital interventions after cancer, even in the clinical trial setting can be poor [20].

Efforts to integrate digital innovations into existing systems of health care have been beset with inefficiency, wasted effort, non‐adoption and abandonment [21]. Greenhalgh et al note that innovation success (and failure) can be influenced by complexity at the levels of the illness or condition, the technology, the value proposition, the adopter system (professionals, patients and caregivers), the organisation(s), the wider context (institutions and society) and how these domains interact and evolve over time [22]. Importantly, each domain involves human behaviours and interactions, meaning that identifying and engaging with key stakeholders at all stages of the design and development processes are likely to be crucial to the success of any digital intervention [23].

We set out to co‐design a digital health intervention [24] to address unmet needs and improve outcomes in people who have completed potentially curable treatment for cancer. There were not many preconceived ideas about the nature of the intervention. We placed no limitations on the setting of the intervention (e.g., hospital‐based, self‐led at home), the format (e.g., web‐based, app or electronic health record enhancement) or the type of task (e.g., symptom management support, administrative function, intervention to enhance patient‐professional communication). We wanted the intervention to be used by patients to address a genuine problem or unmet need. The core aim was to involve a wide range of stakeholders in fully describing the current problems and gaps in cancer aftercare that might be addressed by digital solutions and to co‐design an intervention with these stakeholders.

The aim of this paper is to summarise results from the co‐design workshops and to describe how the workshop informed the core components of the Structured Personalised Assessment for Reviews after Cancer (SPARC) tool.

## 2 Materials and Methods

### 2.1 Study Design and Procedures

The Medical Research Council (MRC) framework for developing complex interventions recommends seeking ‘meaningful engagement with appropriate stakeholders’ [25] but does not stipulate how. This study used principles of accelerated experience‐based codesign (EBCD) [26] and the ‘future workshop’ approach [27]. These approaches have been shown to work well together in participatory health research [28].

Two co‐design workshops were held, each with a wide range of professional and patient stakeholders [27]. A full description of the participant backgrounds in each focus group is detailed in Data File S1. The ethos of the workshops was that every participant would be treated as an equal partner in the research process. Participants were greeted personally on arrival by a member of the research team. Both workshops started with a welcome and introduction from the lead researcher, during which participants were introduced to Chatham House rules [29] and told explicitly that they were considered as equal partners in the research process.

Participant comfort was a priority, and participants were reminded that they could take breaks or leave the workshops at any time. Accessible venues were chosen, and regular sustenance breaks gave participants a chance to mix informally and to get to know one another. Experienced facilitators ensured that all participants had a chance to express their views. Talking about cancer can be upsetting. Participants were reminded about sources of support and the research team had written lists of cancer support organisations.

The first workshop was designed to elicit problems, find solutions and prioritise content for a digital intervention. The second workshop was designed to bring a prototype solution back to the stakeholders, refine intervention design and content, and plan for implementation and evaluation.

The project initially focused on prostate and colorectal cancers, which were selected as exemplar cancers to include people who had experienced a wide range of different treatments, symptoms, side effects and follow‐up regimes. The reason for selecting two common but heterogeneous cancers was to capture cancer‐specific problems and to generate broader insights into the delivery of cancer aftercare. After the first co‐design workshop, participants identified problems that were not specific to the cancer site, and the intervention scope was expanded to include individuals who had experienced any type of cancer.

### 2.2 Participants

The inclusion criteria for this study were broad and anyone who had an interest in developing digital systems for cancer aftercare, who could communicate in English and who could give informed consent, was eligible to take part. The research team emailed representatives from medical software development industries, academia/research, computer science, clinicians (oncologists, general practitioners, nurse practitioners, allied health professionals) and third‐sector organisations to share a study flyer and the programme. Snowball sampling was used in which individuals were invited to share the invitation within their own networks. Third‐sector organisations included Cancer Research UK, CLAN cancer support and Prostate Cancer Scotland. Cancer Research UK published the first workshop in their newsletter.

Patients were also recruited from NHS oncology clinics (NHS Grampian) and were eligible to participate if they had been treated with curative intent for cancer (prostate or colorectal cancer for workshop one and any cancer for workshop two). Patients were approached during clinic appointments by their oncology clinicians and handed a study pack. They were invited to respond directly to the research team if they were interested in participating. Patients were also invited to bring a caregiver or family member.

All participants from the first workshop were invited back to the second workshop and new participants were approached for the second workshop. Invitations to workshop two were also shared with University of Aberdeen's Institute of Applied Health Science Patient Public Involvement (PPI) group.

The study had broad inclusion criteria but it was beyond the scope of this study to focus on palliative care. People receiving palliative care often have different treatment aims and different pathways of follow‐up than those who have been treated for cancer with curative intent. They can also have different groups of professionals involved in their care (e.g., palliative care nurses, hospice teams, etc.).

### 2.3 Co‐Design Workshop One

The first workshop was held in January 2020 in a city centre hotel in Aberdeen. ‘Catalyst films’ were shown at the start of the day [26]. In EBCD, films are produced with stakeholders specifically for the event. In the accelerated approach used in this study, short catalyst films were selected from the ‘living with and beyond cancer – check‐ups’ section of healthtalk.org that were in the public domain [30]. One film depicted an 81‐year‐old male who spoke about worries he had experienced during prostate cancer follow‐up. The second film depicted a 46‐year‐old female who described her experiences of healthcare after colorectal cancer and Hodgkin's lymphoma. Films were chosen that would promote discussion and debate.

The first workshop combined whole group sessions with focus groups. Focus groups were deliberately mixed to include participants from different disciplinary backgrounds and a mix of patients/caregivers and professionals (see Data File S1). There were three focus groups and each group met three times over the course of the day (each for 1‐h sessions, total 3 h per focus group). The three sessions covered the following questions (in the order they were tackled by the groups):

1. What is going well with cancer aftercare, what are the challenges and what could be improved?
2. What information do patients and professionals need to share with each other during cancer follow‐up?
3. Design a digital intervention to improve cancer aftercare: What would it do, and what would be your priorities for the intervention?

Each group made paper notes. The groups were facilitated by R. A., S. M. and L.L. In the third focus group, laminated cards were provided that listed components of existing digital interventions [31] (e.g., ‘book or change appointments, access treatment or care summaries, computerised symptom diary, healthy living support’). Blank cards were also provided so that participants could write their own tasks for digital technologies. The cards were used, if necessary, to prompt discussion, generate ideas and to set priorities about intervention content.

The subsequent stages of the co‐design study were put on hold during the COVID pandemic. The ethics committee and National Health Service Research and Development (NHS R&D) approved restarting the study in December 2023.

### 2.4 Participant Public Input

A prototype intervention was created in Microsoft PowerPoint to illustrate intervention content and wording. Before workshop two, this prototype was shown to people who had a history of cancer from the University of Aberdeen's Patient Public Involvement (PPI) group. Their input was used to gauge the desirability of the proposed intervention and to refine wording of patient facing content before the workshop. Updated versions of the prototype were created following each of these meetings.

### 2.5 Co‐Design Workshop Two

Co‐design workshop two was held in March 2024 in a city centre hotel in Aberdeen (United Kingdom). The workshops started with a whole room discussion before splitting into three focus groups, each 1 h long (see Data File S1), followed by another whole room discussion session. Workshops were facilitated by R. A., A. K. and P. M. The aims of workshop two were:

1. To discuss the results from workshop one, to check that these remained salient and to revisit what is going well in cancer care, the challenges and the current environment in which cancer care is being delivered.
2. To present a prototype of the proposed digital intervention in Microsoft PowerPoint and to gain feedback on the concept, content, format, length, wording and any omissions.
3. To discuss barriers to integrating the proposed intervention into current care pathways including and not limited to data protection and security, digital literacy and inequalities, approvals systems and integration with current electronic systems.
4. To discuss facilitators to intervention success, including examples of good practice or patient‐facing digital systems that are working well in clinical practice, the potential value of the intervention to different stakeholders and design features that would promote engagement.

### 2.6 Data Management and Analysis

All focus groups were audio‐recorded and transcribed verbatim. Workshop one was professionally transcribed. Transcribing software had improved between the workshops, and workshop two was transcribed automatically using Microsoft Word and manually checked for accuracy by L. D.

Written materials from the workshops were photographed or scanned, and the content was summarised into a Microsoft Word document by the lead researcher (R. A.).

Transcripts were read in full by R. A. and L. D. L. D. created summary notes, highlighting key messages from the workshops. R. A. imported transcripts into NVivo version 12 and coded these line‐by‐line using a conventional content analysis approach in which statements were grouped and summarised according to their main messages [32]. Coding categories were derived directly from the data [32]. The essence of the research involved transforming participant experiences, ideas and practical suggestions into a practical intervention. Content analysis provided a flexible and pragmatic method through which key ideas and practical points raised by participants could be synthesised and summarised from the data for that purpose.

### 2.7 Ethics

The study was approved by Yorkshire and The Humber—Leeds East Research Ethics Committee, reference 19/YH/0291 and received NHS R&D approval (NHS Grampian). All participants received a study information sheet in advance of the workshop and provided written informed consent on the day of the workshop. Travel expenses were offered to participants, but no payment was given for participation.

## 3 Results

### 3.1 Participants

The 2 events were attended by 43 people in total of whom 7 were members of the research team/study authors (2 General Practitioners (GPs); 2 Consultant Oncologists; and 3 researchers). There were 26 attendees at the first event (including 5 research team members) of whom 15 were female and 11 male, and 23 attendees at the second event (including 6 research team members), of whom 13 were female and 10 were male. Six participants attended both events.

Participants were computer scientists/information technology/digital health experts (*n* = 9); patients (*n* = 7, including prostate cancer (*n* = 2), colorectal cancer (*n* = 2), lung cancer (*n* = 1), pancreatic cancer (*n* = 1) and undisclosed/missing data for cancer site (*n* = 1); researchers (*n* = 6); Consultant Oncologists (*n* = 4), GPs (*n* = 4); Consultant clinicians with interests in eHealth (*n* = 3); NHS Cancer or service improvement staff (*n* = 3); designers (*n* = 2); allied health professionals (*n* = 2); specialist nurses (*n* = 2) and a caregiver for a patient with myeloma. Many of the participants had more than one role, for example, having had a previous cancer diagnosis or having been a caregiver for somebody with cancer but attending in a professional capacity.

### 3.2 Results of Workshops: Content Analysis

#### 3.2.1 What Is Going Well in Cancer Care? Access to Human Experts and Relationship‐Based Care

Cancer is a highly personal experience and both patients and professional participants spoke about the desire to have cancer follow‐up that is tailored to the needs of the individual and that moves away from a ‘one size fits all’ approach in terms of the structure, content and timing of consultations after cancer treatment. Throughout both workshops, it was apparent that having access to trusted specialist nurses and other experts both during and after treatment was crucial. Relationship‐based care was highly valued by all.

It's just such a personal thing, you know, getting cancer, it's not like getting a cold, or breaking your leg. You know you're going to get better from that. You do not know at that point of diagnosis that you are going to get better, or not, and you really need someone that you can latch onto, go to for help, (…) continuity of care is without value (…) I went through treatment without telling anybody that I had cancer at all, so though I'm an optimistic person, I'm a very private person, (…) I had, if you like, a small team of trusted confidants within the NHS that I could rely on. Patient, history of colorectal cancer, female, workshop one

Patients and professionals described the importance of marrying technical and disease‐based therapies with holistic care of the individual. The multidisciplinary team was a key component of successful cancer aftercare.

I was really surprised, pleasantly surprised that in effect I've got at one end of the spectrum, a consultant who knows how to cut me open, and an oncologist who knows how to poison me in a productive way, I've got a clinical nurse specialist [to] make sure that I understand what's going on and, (…), and then I'm fed onto cancer support, and [they] provide me with counselling and reflexology (…) it's a spectrum where you have at the one end, the hard NHS science driven, evidence driven, medical approach and at the other hand, you still, in that envelope, in that care pathway, you have the very soft psychosocial stuff. Designer, male, attending in professional capacity and speaking about his personal experiences of an upper gastrointestinal cancer, workshop one

Several participants highlighted that any technological innovation must enhance the care delivered by humans rather than replace personalised care by clinicians.

I think that the nurses would pick up if someone is getting really excited about [a problem] or getting panicky about it (…) So, taking out the human element I think is not good, (…) these specialist nurses were absolutely great (…) people in the support group had to [contact them] and they said that they calmed them down. They were: ‘fine, no, what you're experiencing is yes, you're expected to experience that, but tomorrow it will be OK’. So, if we do everything by technology that gets lost. Carer, female, workshop two

#### 3.2.2 What Aspects of Cancer Aftercare Could Be Improved? Support From Primary Care for On‐Going Problems

Patients felt well supported during cancer treatment and hospital‐based follow‐up, although some participants suggested that routine advice about diet, exercise and signposting to third‐sector organisations was inconsistent.

Being discharged from hospital care could feel like ‘falling off a cliff’ (a metaphor that was used by several patient and professional participants). Patients were uncertain about whether general practices would or should provide any routine follow‐up or monitoring. Participants noted that cancer and its treatments influenced their appraisal and interpretation of new symptoms. Having access to a professional to reassure (or investigate) was highly valued.

What happens with cancer is it shatters your confidence in yourself in terms of, we all think we know our bodies, but actually [cancer] happens and (…) therefore, every problem that happens afterwards initially is reflected back to cancer. Consultant Oncologist, male, workshop two

Multimorbidity management was acknowledged as an important aspect of holistic cancer aftercare that went beyond the remit of the oncology service. Specialist nurses managed cancer‐specific problems and sign‐posted patients with other health problems to primary care.

Remember, a lot of these patients are living with other conditions, so sometimes the cancer's actually not the most significant disease that they have. Specialist prostate cancer nurse, male, workshop one

However, there was a sense amongst patients and some hospital‐based clinicians at both workshops that general practices were difficult to access and under‐resourced. An oncologist described feeling ‘guilt’ when he directed patients to make an appointment with their GP for issues such as hormone replacement therapy after cancer treatment. Patients also felt like their GPs were too busy to contact about non‐urgent concerns, worries or more chronic symptoms.

My GP, as somebody says, has no idea how I am, what I'm experiencing and there are things that you do experience after 10 years (…) but nobody's asking me about it, nobody seems to be interested in it (…) and it seems very minor to actually get in touch with the GP and say ‘look there are a number of niggles I've got, would you have time to see me?’ Because you know they wouldn't have time or they might, but it's not… it's wasting [their] time, when they could be doing something better. But it's niggling at me, and nobody's asking my opinion. Patient, history of lung cancer, female, workshop two

The issue of lack of time and capacity in primary care was raised independently by different groups. One experienced GP from a semi‐rural general practice (co‐design day one) responded by suggesting that ‘it's hard to find the time, but you can make it, and it's about how GPs as individuals can make that time when it's needed’. The involvement of GPs in providing on‐going care for their patients beyond cancer treatment was a desirable and necessary component of high‐quality cancer aftercare. Patients and professionals from both workshops highlighted that continuity of care was decreasing in general practice and that this was a threat to high‐quality cancer care.

In my view, that's the GP's job, our job is to help you understand what's happening to you, and help negotiate your way through things, which perhaps is maybe something that's getting harder to do with the lack of continuity sometimes. Continuity makes that much easier. GP, male, workshop one

There was a sense that a digital intervention should involve primary care, should promote informational continuity and encompass transitions in care.

when somebody finished treatment, there's that kind of transition, where, as you say, the anxieties, (…) mean people are more honing in on secondary care, where ultimately their long term care is going to be their primary care team, because all the other health problems that we're going to have in our life are going to happen whether we've had cancer or not, and so we mustn't disenfranchise primary care (…) I like the idea that digital might allow us, even does now, [to] have much more of a kind of continuity with the primary care team knowing what's going on. Consultant Oncologist, male, workshop one

#### 3.2.3 Implementing Digital Technologies Into Cancer Aftercare

It was broadly acknowledged across both workshops that implementing digital interventions into any existing clinical pathway would be challenging. Participants brought their own examples of digital and non‐digital innovation efforts that had failed to be implemented or adopted. Participants mentioned a range of barriers to innovation, including variability in clinical pathways, different approvals procedures and requirements between different health boards, ‘risk averse’ information governance processes, and the lack of capacity within the NHS to manage, reach consensus about and effect change. There was frustration that existing systems with proven benefits were not being routinely implemented across Scotland.

I sit in the cancer task force (…) we sit there with [senior Scottish Government minister] (…) however many health boards there are they're all working in different ways (…) there's trials going on, there's good practise being identified, but you can't get it put into all of the different health boards because they all have their own way of working. And that's the frustrating thing, because if there is something working you know well, why wouldn't you adopt it across the whole country? It's not a big country (…). Patient, history of lung cancer, female, workshop two

Participants recommended that a new intervention should not ‘reinvent the wheel’. The intervention should take advantage of and could be integrated into existing infrastructure, digital systems and processes of care wherever possible.

The intervention would have to operate within a system that is struggling to meet demands and has limited capacity. The intervention should not create additional work and should be able to demonstrate a value proposition to those using it (both patients and clinicians). Participants stressed that if a digital system collects information from a patient, the information must be acted upon.

We work in a service with cancer. We're prioritised. When I look at what happens in terms of [long waits for] imaging and things, who knows what's happening to people who've got benign disease (…) Mental health [is] like a desert. (…) nothing worse than asking people to do things and then nothing happens at the end of the line, that must be so demoralising (…) it's sort of saying we care about you but then you realise, actually they don't care enough, they've not actually organised the service properly. It's like a negative thing. Consultant Oncologist, male, workshop two

Participants highlighted ways that digital technologies could save the clinician's time, for example, by identifying those who do not have problems, by automatically sign‐posting to self‐management and third‐sector resources, by collecting structured information before consultations that help patients and clinicians to prioritise problems and by automating some tasks (e.g., scoring patient‐reported outcome measure questionnaires).

#### 3.2.4 A Potential Solution: Integrating Cancer Reviews Into Primary Care Multimorbidity/Chronic Disease Management (CDM) Activities and Using Digital Technologies to Create Capacity

Instead of replacing in‐person care, there was an ambition that technology could be used to automate some aspects of follow‐up to create more capacity for clinicians and patients to spend time together when necessary.

You're saying you can no longer see a nurse (…) the questions in my head are: why is that? Is it simply funding cuts, the nurses don't exist? Or is it the level of demand? And what are those nurses doing and are they spending time, you know, scoring a questionnaire that you filled out and actually a computer could score that questionnaire and will save time. It's what can we do that releases capacity so that when you need to speak to someone you can speak to someone. NHS service improvement manager, female, workshop two

Another ambition was for technology to standardise and improve the breadth of problems dealt with during cancer aftercare. Many patient problems and needs were predictable. For example, in prostate cancer follow‐up, specialist nurses asked all patients about similar problems.

I ask just about everybody the same questions to start with, you know, about their energy, their hot flushes, any bowel symptoms, any bladder symptoms, (…) the core things that are automatically, I ask everybody that, regardless. Specialist nurse practitioner for prostate cancer, female, workshop one

One potential solution was for cancer aftercare to be considered more in terms of the CDM that routinely takes place in primary care. One computer scientist who had diabetes compared cancer follow‐up to diabetes care:

Computer scientist: Diabetes care has a kind of a calendar, so I have things like retinopathy, foot test (…) I feel monitored (…)

Oncologist: Well, they follow you up forever don't they with diabetes

Computer scientist: Around this time, I receive a letter for a foot test, not much, but it just gives me kind of reassurance (…) and that helps actually I feel connected with the care. (…)

Oncologist: But I think that's the difference that the system is committed to following up diabetic people forever (…) nobody gets chucked out

Computer Scientist: Yeah, it's a chronic thing

Oncologist: and that's what's missing in cancer

### 3.3 Operationalising the Structured Personalised Assessment for Reviews After Cancer (SPARC) Intervention

The results of the codesign days directly informed a new intervention that has been designed to enhance review appointments between patients and their primary care clinicians. The co‐design results and their influence on intervention development are summarised in Table 1. Co‐design directly informed the setting of the intervention (primary care), the scope, purpose and value proposition to patient and health system, the intervention content (structured, comprehensive but brief clinical review) and the mode of deployment and implementation (asynchronous digital consulting).

**Table 1:** How co‐design shaped intervention components.

Key finding from co‐design event	Impact on design of SPARC intervention
Hospital cancer care works well but patients have unmet needs that are best addressed in primary care	Intervention based in primary care and could standardise high‐quality reviews
Human relationship‐based care is central to good cancer aftercare—Technology should enhance human interactions and not replace them	The intervention is used to structure cancer reviews as part of existing Quality and Outcomes Framework in England and integrated into other chronic disease management reviews (e.g., diabetes, asthma, etc.)
There is variation in care, particularly around support with diet, exercise and other predictable problems	Existing best practice guidelines used to structure the tool under comprehensive domains Automated sign‐posting to a range of evidence‐based resources, based on participant responses
Don't reinvent the wheel	Simple headings, structured from existing evidence syntheses and guidelines Simple information technology platform that can work with existing practice information technology systems or be adapted for use with any practice system Tool operationalised with industry partner
Use digital technologies to create capacity	Ability to identify people who don't require an in‐person cancer care review Automated sign‐posting to useful resources outside the National Health Service Intervention appropriate for use by any primary care clinician for example, pharmacist, nurse, doctor in training Patient concerns are prioritised before consultation

The SPARC tool aims to improve and standardise the support offered to cancer survivors in primary care and could be used in general practices to structure reviews that already take place as part of the Quality and Outcomes Framework in England or integrated within other CDM reviews (e.g., diabetes, asthma, hypertension) in primary care. The tool could also be particularly useful at transition points, following completion of treatment or discharge from hospital care.

Asynchronous consulting is becoming widespread in primary care, and many practices already use electronic systems that can search their electronic health records, identify patients with specific conditions and send automated text messages or emails to patients with links to structured clinical reviews [33, 34, 35].

A full protocol describing the intervention and how it will be evaluated will be published separately. Briefly, participants with a history of cancer will be invited to complete a short electronic questionnaire that is structured according to an International Consensus Framework [36], ensuring comprehensive coverage of core domains of cancer survivorship care [36], around 2 weeks before a planned cancer care or CDM review appointment in primary care. Questions relate to prevention and surveillance for recurrence and new cancers, surveillance and management of physical effects, psychosocial effects and chronic medical conditions, and health promotion and disease prevention.

A prominent message from the workshops was: ‘don't reinvent the wheel’. To this end, the intervention will be operationalised with an industry partner [33] with a technology that has been widely adopted already across UK primary care [33]. The industry partner has already tackled some of the technical, electronic health record integration challenges and information governance issues [33]. The full content of SPARC will be made freely available, so that it can be adapted to be used with any asynchronous primary care consulting system.

Responses to the questionnaires will be used to automatically sign‐post patients to existing evidence‐based resources (e.g., services offering support with exercise, cancer financial support and on‐line symptom management resources). Patients will be able to indicate whether a formal review in primary care is desirable, and their preferred mode of review (e.g., telephone, in person). Where multiple problems are identified, participants will be asked to prioritise their problem(s). The feasibility, intervention content, perceived value (to patients and clinicians) and influence on the primary care consulting process will be evaluated in around six general practices in subsequent research.

## 4 Discussion

We co‐designed the SPARC digital intervention through a process of stakeholder engagement and collaboration to address some of the key problems articulated as being experienced by those living beyond cancer. Specifically, the intervention targets primary care, considers cancer in the context of other chronic diseases, and uses digital technologies to standardise and structure a review that is brief but comprehensive in the range of issues covered. SPARC is designed to enhance rather than replace human contact.

In England, the Quality and Outcomes Framework (QOF) contract guidelines suggest that patients should be contacted within 3 months of a cancer diagnosis to offer pro‐active support. SPARC could be used to structure these reviews, but we found that patients particularly need primary care support after their treatment has finished and around the point of hospital discharge.

The ambition for SPARC is that it is integrated into routine chronic disease management reviews for those with a history of cancer. Multimorbidity is becoming the norm, particularly in older individuals, and cancer is a disease associated with ageing [37]. Conducting brief structured cancer care reviews alongside care for other chronic diseases could be one way of optimising the role of primary care in cancer aftercare. These reviews could be particularly helpful at transition points in care but could also continue in the long‐term to identify late effects of treatment and to support healthy behaviours.

Interestingly, in a recent interview study with 6 caregivers and 35 patients who had received potentially curative treatment for prostate and colorectal cancer, most considered their cancer in terms of a ‘discrete episode’ that had been cured [38]. For example, a participant with hypertension had altered his diet, lifestyle and did regular monitoring to manage hypertension but made few adjustments after colorectal cancer treatment. There could be advantages of re‐framing cancer as a chronic disease, particularly when many participants in this study had lasting effects of cancer and when behavioural interventions such as exercise, smoking cessation and dietary changes could confer survival benefits [39].

Participants' descriptions of ‘falling off a cliff’ correspond to the concept of ‘abandonment’ after hospital discharge that has been described in the broader cancer literature [7, 40]. Our research suggests that more needs to be done in primary care to improve experiences and outcomes in those living beyond cancer. This is a difficult message in the context of crisis in the NHS [41, 42]. Limited capacity in primary care is likely to be the greatest threat to implementing this or any innovation or new intervention. It is essential to evaluate the feasibility and influence on costs of care and routine workload of adding brief cancer reviews to chronic disease management activities in subsequent evaluations.

Barriers and facilitators to implementing and scaling digital interventions have been well characterised [43]. Greenhalgh and colleagues framework for theorising and evaluating nonadoption, abandonment, scale‐up and spread (NASS) [22] gives important insights into key considerations. In this study, a potential enabler to success is that practices are already pro‐actively adopting digital asynchronous consulting models for chronic disease management reviews. ‘Medlink’ is currently available to over 2 million primary care patients in the United Kingdom [33].

Recent research, including local research in NHS Scotland, suggests that asynchronous consulting models are feasible and acceptable to patients and clinicians [44]. One systematic review identified 19 studies of asynchronous consulting and found that clinical outcomes from ‘e‐visits’ were comparable to in‐person visits and that healthcare costs were reduced for some conditions [45].

Considering the NASS model, it will also be important to work with cancer organisations and political/policymakers to make the case for cancer reviews to be included within the remit of chronic disease management.

### 4.1 Strengths and Limitations

Key strengths of the co‐design study include the rich data that were generated from in‐depth discussions over two workshops, the inclusion of stakeholders with a wide range of different disciplinary backgrounds with varying experiences and perspectives, and the robust processes in place to manage and analyse qualitative data.

A limitation is the delay between the two workshops and the fact that many of the original participants in workshop one were not able to be contacted and/or recruited again for workshop two. Detailed participant demographics were not collected at the event. There was a trade‐off between making participants feel at ease, completing research paperwork on the day (particularly written consent forms) and collecting formal demographic information from participants. Given that patients were equal partners, we would not have collected information (e.g., about ethnicity, age, socioeconomic status) only from patients but would have required this information from all participants. It is possible that certain participants are under‐represented in our study, and in subsequent stages of evaluation, it will be crucial to include participants from a diverse range of backgrounds.

Co‐design shaped almost every aspect of intervention design, from the setting and content to the implementation. However, it is important to reflect on the extent to which any funded project of work can truly achieve equal partnership between the budget holders and other stakeholders. Quality improvement projects require strategic management and involve multiple small decisions (e.g., event venue, catering options) and larger decisions (e.g., the company that will programme the intervention). In the research setting, these decisions are ultimately taken by the Principal Investigator and will be limited by individual project resources. Co‐design is an important step forward in terms of sharing power with stakeholders, but it is important to acknowledge that structural limitations still exist.

## 5 Conclusion

Patients experience predictable problems after completion of potentially curative treatment for cancer, and there is a requirement for on‐going primary care input after discharge from hospital. There is a case for cancer to be considered as a chronic disease and for general practices to offer regular reviews to those with a previous cancer diagnosis. We co‐designed a brief structured assessment that could help general practices to integrate high‐quality cancer survivorship reviews into routine care. The intervention will now be evaluated for feasibility and desirability with patients and in general practices.

## Author Contributions

**Rosalind Adam:** conceptualisation, investigation, funding acquisition, writing – original draft, methodology, writing – review and editing, formal analysis, project administration, data curation, supervision. **Lisa Duncan:** investigation, writing – review and editing, project administration, formal analysis. **Sara MacLennan:** writing – review and editing, data curation. **Louise Locock:** writing – review and editing, data curation. **Anne E. Kiltie:** writing – review and editing, data curation. **Leslie Samuel:** writing – review and editing, data curation. **Peter Murchie:** writing – review and editing, data curation.

## Conflicts of Interest

The authors declare no conflicts of interest.

## Supporting information

Supporting information.

## Data Availability

The data generated during this study are not publicly available because participants did not consent to data sharing. The authors would be happy to share relevant anonymised excerpts from transcripts/longer quotations on reasonable request. Raw data in the form of participant quotations are included throughout this paper to illustrate the key points.
