# Supplementary material for: Co‐Design of the Structured Personalised Assessment for Reviews After Cancer (SPARC) Intervention
Source: Health Expect. 2025 Feb 6;28(1):e70174. doi: 10.1111/hex.70174 (PMC11802641; doi:10.1111/hex.70174)
Supplement: Supplementary file 1 — Supporting information. [file HEX-28-e70174-s001.docx]

**Workshop one (n=26)**

| **Group 1** | **Group 2** | **Group 3** |
| --- | --- | --- |
| Researcher | Researcher | GP |
| Consultant Oncologist | Consultant Oncologist | Consultant Oncologist |
| Computer scientist/IT specialist | Specialist nurse | Researcher |
| Specialist nurse | Computer scientist/IT specialist | GP |
| GP | Designer | Researcher |
| Patient | Consultant clinician eHealth/innovation | Cancer support |
| Computer scientist/IT specialist | Cancer coordinator | Computer scientist/IT specialist |
| Researcher | Patient | Caregiver |
| Allied Health Professional | GP |  |

**Workshop two (n=23)**

| **Group 1** | **Group 2** | **Group 3** |
| --- | --- | --- |
| Consultant Oncologist | GP | GP |
| Researcher | Consultant Oncologist | Computer scientist or IT specialist |
| Computer scientist/IT specialist | Allied Health Professional | Researcher |
| Computer scientist/IT specialist | Computer scientist or IT specialist | Computer scientist or IT specialist |
| Computer scientist/IT specialist | Designer | Programme manager |
| Consultant Clinician | Patient | Caregiver |
| Patient | Patient | Patient |
| Patient | Consultant clinician eHealth/innovation |  |
